# Interleukin 6 and Rheumatoid Arthritis

**DOI:** 10.1155/2014/698313

**Published:** 2014-01-12

**Authors:** Yuji Yoshida, Toshio Tanaka

**Affiliations:** ^1^Department of Respiratory Medicine, Allergy and Rheumatic Diseases, Osaka University Graduate School of Medicine, Osaka 565-0871, Japan; ^2^Department of Clinical Application of Biologics, Osaka University Graduate School of Medicine, 2-2 Yamada-oka, Suita City, Osaka 565-0871, Japan; ^3^Department of Immunopathology, WPI Immunology Frontier Research Center, Osaka University, Osaka 565-0871, Japan

## Abstract

Interleukin-6 (IL-6) is a representative cytokine featuring pleiotropic activity and redundancy. A transient synthesis of IL-6 contributes to host defense against infectious agents and tissue injuries by inducing acute phase reactions and immunological and hematopoietic responses. However, uncontrolled persistent production of IL-6 may lead to the development of several immune-mediated diseases. Rheumatoid arthritis (RA) is a chronic disease with joint and systemic inflammation resulting from immunological abnormalities and it has been found that IL-6 plays a key role in the development of this disease. Clinical trials in various parts of the world of tocilizumab, a humanized anti-IL-6 receptor antibody, have proved its efficacy and tolerable safety either as monotherapy or in combination with disease-modifying antirheumatic drugs. As a result, it is currently used as a first-line biologic for the treatment of moderate-to-severe RA in more than 100 countries. Clarification of the mechanism(s) through which tocilizumab exerts its effect on RA and of the reason(s) why IL-6 is continuously produced in RA can be expected to lead to the best use of this agent for RA patients and aid in investigations into the pathogenesis of RA.

## 1. Introduction

Rheumatoid arthritis (RA) is characterized by synovial inflammation and hyperplasia, autoantibody production such as rheumatoid factor (RF) and anti-citrullinated protein antibody (ACPA), cartilage and bone destruction, and systemic features, including cardiovascular, pulmonary, psychological, and skeletal disorders [1]. Although its exact pathogenesis remains to be determined, a multistep progression is considered for the development of RA [1]. First, environment-gene interactions promote loss of tolerance to self-antigens that contain a citrulline residue generated by posttranslational modification. Second, the anticitrulline response is induced in T cells as well as B cells. Thereafter, localization of the inflammatory response occurs in the joint and synovitis is initiated and perpetuated by positive feedback loops and promotes systemic disorders. In this process, various cells and their products contribute to the development. For instance, as key molecules many cytokines including TNF-*α*, IL-1, IL-7, IL-15, IL-17A, IL-17F, IL-18, IL-21, IL-23, IL-32, and IL-33 are implicated in the pathogenesis of RA [1].

Before this century, the only drugs available for RA were nonsteroidal anti-inflammatory drugs (NSAIDs), corticosteroids, and disease-modifying antirheumatic drugs (DMARDs) including gold, chloroxine, salazosulfapyridine, and methotrexate (MTX). However, these drugs were often not effective enough to completely suppress disease activity and joint destruction. The arrival of biological agents (biologics, biological DMARD) such as TNF inhibitors, abatacept, an inhibitor of T-cell costimulation, and rituximab, an agent leading to B-cell depletion induced a paradigm shift in the treatment of RA and Treat-to-Target (T2T) treatment proved to be successful for disease remission and protection against joint destruction [2].

Dysregulated persistent production of interleukin-6 (IL-6) also plays a key role in the development of the main characteristics of RA [3–5]. In response to the supposition that IL-6 targeting could be a novel therapeutic strategy for RA, a humanized anti-IL-6 receptor monoclonal antibody (Ab), tocilizumab (TCZ), was developed. Subsequent clinical trials conducted all over the world have proved the efficacy and tolerable safety of TCZ and it is currently used as an innovative biologic for the treatment of RA in more than 100 countries. Moreover, TCZ was also approved for the treatment of systemic juvenile idiopathic arthritis in Japan, USA, EU, and India, and Castleman's disease in Japan and India, while recent various case reports or pilot studies of off-label use with TCZ suggest that it is widely applicable for the treatment of other immune-mediated diseases including vasculitis syndrome, adult-onset Still's disease, systemic lupus erythematosus, or others [4, 5]. In this paper, we present current evidence of the pathological role of IL-6 in the development of RA and the efficacy and safety profile of TCZ for RA and discuss future aspects of IL-6 targeting strategy for RA.

## 2. IL-6 and Signaling Pathway of IL-6

IL-6 is a glycoprotein with a molecular weight of 26 kDa and pleiotropic activity. It was first identified as B cell differentiation factor (BCDF) or B cell stimulatory factor 2 (BSF-2), which is a T-cell-derived soluble factor that induces the differentiation of activated B cells into Ab producing cells [6, 7]. Complementary DNA of IL-6 was successfully cloned by Hirano et al. in 1986 [8] and the resultant molecule was found to be identical to hybridoma growth factor (HGF), which derives its name from its promotion of growth of fusion cells with myeloma, to hepatocyte-stimulating factor (HSF) with its promotion of synthesis of acute phase proteins such as C-reactive protein (CRP), serum amyloid A (SAA), haptoglobin, fibrinogen, and hepcidin in hepatocytes, or to interferon (IFN)*β*2 due to its IFN anti-viral activity [9–11]. Subsequent studies also revealed that IL-6 performs multiple and essential functions in immune regulation, inflammation, and even oncogenesis and could be a key mediator for the development of many chronic inflammatory or autoimmune diseases including RA [12–14].

**Figure 1:**
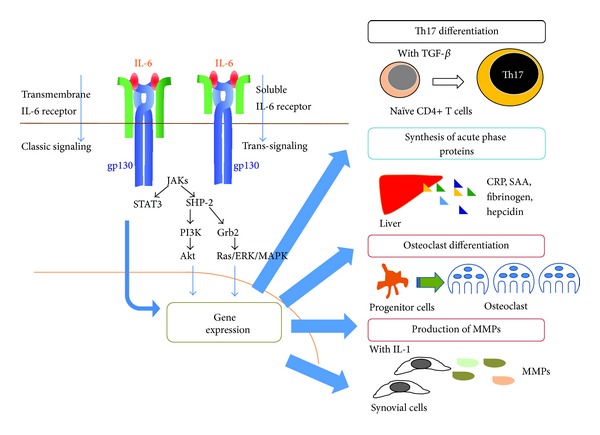
IL-6 exerts its pleiotropic activity by activation of gp130 through its binding to transmembrane or soluble IL-6 receptor. IL-6 initiates the IL-6 signaling pathway through binding to transmembrane or soluble IL-6 receptor. The resultant complex then induces homodimerization of gp130, which leads to activation of a signaling system. Transcriptional factors including STAT3 activate various gene expressions, resulting in cell differentiation or proliferation. JAKs: Janus kinases; STAT3: signal transducer and activator of transcription 3; SHP-2: SH2 domain-containing tyrosine phosphatase 2; PI3K: phosphoinositol-3 kinase; Grb2, growth factor receptor-bound protein 2; ERK: extracellular signal-regulated kinase; MAPK: mitogen activated protein kinase; Akt: protein kinase B; TGF-*β*: transforming growth factor beta; CRP: C-reactive protein; SAA: serum amyloid A; MMPs: matrix metalloproteinases.

IL-6 triggers its signaling system through binding to an 80 kDa transmembrane IL-6 receptor (IL-6R) (Figure 1) [15, 16]. After binding to IL-6R, the complex consisting of IL-6 and transmembrane IL-6R associates with signal-transducing molecule gp130, resulting in the activation of downstream signaling events via Janus kinase (JAK) in target cells [17–20]. This activation is known as classic signaling pathway. Transmembrane IL-6R is expressed on only limited cells such as hepatocytes and some leukocytes, whereas gp130 is expressed on various cells. A soluble form of IL-6R (sIL-6R) lacking the cytoplasmic region exists in serum and has a similar affinity to IL-6 as transmembrane IL-6R. The complex of IL-6 and sIL-6R can also bind to gp130, leading to the activation of signaling cascade. This process is called trans-signaling. Accumulating evidence suggests that IL-6 trans-signaling is proinflammatory, whereas classic signaling is needed for regenerative or anti-inflammatory activities [21].

JAK is a member of the tyrosine kinase family, and its phosphorylation further induces the activation of signal transducer and activator of transcription (STAT) 3 and hyperphosphorylation of mitogen-activated protein kinases (MAPKs) [22]. The activation of the former is dependent on phosphorylation at tyrosine 759 (Y759) in gp130 and the latter requires phosphorylation on any residues of Y767, Y814, Y904, and Y915, which are all encountered in the YXXQ motif context. STAT3 then stimulates the expression of several genes leading to the induction of cell growth and differentiation [23–26]. MAPK also activates several transcription factors associated with acute phase protein synthesis and cell growth. Phosphorylation of a phosphoinositol-3 kinase (PI3K) by JAK results in activation of a third pathway by IL-6, which is the PI3K protein kinase B (PkB)/Akt pathway [27]. The activated Akt then phosphorylates several downstream targets to upregulate cellular survival [28].

## 3. Pathological Role of IL-6 in RA

RA is a chronic, progressive inflammatory disease of the joints and surrounding tissues accompanied by intense pain, if untreated, irreversible joint destruction, and systemic complications such as fatigue, anemia, and fever [1]. RA patients typically show immunological abnormalities leading to the production of autoantibodies such as RF and ACPA.

IL-6 has been shown to contribute to the production of autoantibodies by acting on plasmablasts [29]. Historically, IL-6 was originally identified as a helper T-cell-derived soluble factor that promoted immunoglobulin secretion by activated B cells [6, 7], while recent findings indicate that IL-6 also acts as regulator of CD4+ T cell differentiation and activation. IL-6 signaling has been found to control proliferation and resistance of resting T cells against apoptosis by promoting IL-2 production and STAT3 activation. In addition, IL-6 influences T cell effector functions by promoting Th2 cell differentiation through upregulation of nuclear factors of activated T cells (NFAT)c2 and c-maf, while it blocks IFN-*γ*-signaling and inhibits Th1 cell differentiation [30]. Moreover and more important, in the presence of transforming growth factor (TGF)-*β*, IL-6 is able to promote Th17 cell differentiation through STAT3-mediated upregulation of retinoid orphan receptor (ROR)*γ*t, while it inhibits TGF-*β*-induced regulatory T cell (Treg) differentiation [31, 32]. IL-6 thus promotes predominance of Th17 over Treg in the effector CD4+ T cell subsets, which is thought to play a major role in the development of RA and various other immune-mediated diseases. In addition, IL-6 has been shown to promote T follicular helper cell development, which secretes IL-21, another B cell differentiation factor [33–35].

It has further been demonstrated that IL-6 is involved in local inflammation causing joint destruction by inducing endothelial cells to produce IL-8 and monocyte chemoattractant protein-1 (MCP-1) and to activate expression of adhesion molecules and recruit leukocytes to involved joints [36]. Synoviocytes can produce IL-6, while IL-6 can induce synoviocyte proliferation and osteoclast differentiation through receptor activator of NF-kappa B ligand (RANKL) expression [37, 38]. This stimulation by IL-6 is also associated with the development of osteoporosis and bone destruction. IL-6 and IL-1 synergistically enhance the production of matrix metalloproteinases (MMPs) from synovial cells, which may lead to cartilage and joint destruction [39]. Furthermore, enhanced angiogenesis and vascular permeability of synovial tissue are pathological features of RA resulting from the excess production of vascular endothelial growth factor (VEGF), which is also induced by IL-6 in synovial fibroblasts [40].

Systemic inflammatory signs and symptoms related to RA include fever, malaise, sleep disturbance, muscle weakness, and anemia, while laboratory findings observed in patients with RA are CRP elevation, hypercoagulability, and hypoalbuminemia. These are thought to be mostly mediated by IL-6 [5, 10, 11]. IL-6 induces hepcidin production, which blocks the action of iron transporter ferroportin 1 on gut and thus reduces serum iron and hemoglobin levels [41]. Moreover, RA patients often suffer from thrombocytosis, also mediated by IL-6, which promotes the differentiation of megakaryocytes into platelets [42].

These findings prove that IL-6 plays a key role in the induction of immunological abnormalities and in the development of joint and systemic inflammation of RA.

IL-6 was found to be elevated in serum as well as synovial fluid of patients with RA [43]. These levels correlated with disease activity of RA, while successful treatment with DMARDs or TNF inhibitors has been shown to reduce serum IL-6 concentrations [44–46]. Moreover, reduction in IL-6 levels during the first 12 months of treatment is reportedly a prognostic marker for better clinical outcome [47]. Recently, it was also shown that a decrease in serum IL-6 levels during TCZ treatment can be a predictive marker for maintenance of remission status [48]. These findings clearly point to the pathologic role of IL-6 in RA. However, it remains unknown what the exact mechanisms are through which IL-6 is continuously oversynthesized in RA and TCZ treatment leads to a reduction in intrinsic production of IL-6.

The pathological role of IL-6 in several animal models of RA was also documented. Collagen-induced arthritis (CIA) is the most well-known animal model of RA, in which injection of mice with type II collagen produces an immune response directed at connective tissues. In the CIA model, activated T cells produce augmented amounts of both Th1 and Th17 cytokines, while deficiency of IL-6 activity through gene knockout suppresses Th17 cytokine production and clinical symptoms of arthritis [49, 50]. Similar results have been found for blockade of IL-6 signaling by using an anti-mouse IL-6R Ab [51, 52]. In this model, the proliferative response of B and T cells isolated from lymph nodes of anti-IL-6R-treated mice was significantly suppressed compared to controls. In addition, anti-IL-6R treatment led to amelioration of the histopathological features of arthritis including inflammatory synovitis and joint erosions. IL-6 gene deficiency and blockade of IL-6 activity also reduced severity of arthritis in other mouse models of RA, such as antigen-induced arthritis (AIA), an immune complex model of RA, and SKG mice which spontaneously develop autoimmune arthritis with ageing due to a spontaneous mutation in the zeta-chain-associated protein kinase-70 (ZAP-70) gene [53–57].

## 4. Development of Tocilizumab, a Humanized Anti-IL-6 Receptor Monoclonal Antibody

The findings described above led to the concept that IL-6 targeting might constitute a novel therapeutic strategy for RA. In response to this supposition, TCZ, a humanized anti-IL-6R monoclonal Ab of the IgG1 class, was developed [58]. TCZ blocks IL-6-mediated signal transduction through inhibition of IL-6 binding to transmembrane as well as soluble IL-6R. The first clinical evaluation of the efficacy of TCZ was conducted for the treatment of seven patients with Castleman's disease, a chronic inflammatory disease characterized by multiple lymph node swellings with massive infiltration of mature plasma cells [59]. Such patients present with severe inflammatory symptoms such as high fever, anemia, increased levels of acute-phase proteins, and hyper-*γ*-globulinemia. After TCZ administration, the fever promptly diminished, CRP levels became normalized, and hemoglobin levels increased. The efficacy of TCZ was next proved in a clinical trial using 28 patients with Castleman's disease [60], and this resulted in its approval as an orphan drug for the Japanese market in 2005.

The further development of TCZ entailed phase I and II clinical trials of TCZ for RA performed between 2002 and 2006 with favorable results [61–63]. The first trial was a randomized, double-blind, placebo controlled, dose-escalation trial in the UK [61]. Patients treated with 5 mg/kg or 10 mg/kg TCZ showed significant improvement by week 2. The next dosing determination trial was conducted in Japan. Patients were given a placebo or TCZ (4 or 8 mg/kg every 4 weeks) and 8 mg/kg TCZ resulted in the greatest improvement [62].

## 5. Efficacy of Tocilizumab in Phase III Clinical Trials and Actual as in Clinical Settings

Seven phase III randomized controlled trials (RCT) were conducted to evaluate the clinical efficacy of TCZ as either monotherapy or in combination with DMARDs including MTX (Table 1) [64–70].

### 5.1. Tocilizumab Combination Therapy

For further assessment of the efficacy of TCZ, RCTs of TCZ combination therapy were conducted. The OPTION trial was designed to evaluate the usefulness of TCZ (4 or 8 mg/kg every 4 weeks) in combination with MTX and the results demonstrated that this combination therapy was effective for and well tolerated by patients with active RA and an unsatisfactory response to MTX [64]. The TOWARD study compared the efficacy of TCZ (8 mg/kg every 4 weeks) plus DMARDs with that of DMARDs only for inadequate responders to DMARDs [65], and the RADIATE study compared the efficacy of TCZ (4 or 8 mg/kg every 4 weeks) plus MTX with that of MTX only for inadequate responders to TNF inhibitors [66]. Both studies showed evidence of a significant reduction of disease activity in the TCZ groups. The LITHE trial demonstrated that TCZ (4 or 8 mg/kg every 4 weeks) plus MTX had superior American College of Rheumatology (ACR20), 50 and 70 responses at 52 weeks compared with controls treated with placebo plus MTX [67].

### 5.2. Tocilizumab Monotherapy

The AMBITION trial was designed to compare the efficacy and safety of TCZ monotherapy with those of MTX monotherapy [68]. The results showed rapid improvement in RA disease activity and a favorable risk benefit profile for TCZ compared to MTX monotherapy. The SAMURAI study, which evaluated the efficacy of TCZ monotherapy for patients with an inadequate response to DMARDs, also showed a superior efficacy of TCZ compared to DMARDs [69]. Finally, the SATORI study investigated the efficacy of TCZ monotherapy for moderate-to-severe active RA patients with an inadequate response to low doses of MTX [70]. At week 24, the ACR20 response rate was 80.3% for the TCZ group and 25.0% for the MTX group.

In summary, TCZ as either monotherapy or in combination therapy with MTX or other DMARDs was highly efficacious for RA patients (Tables 1(a) and 1(b)).

### 5.3. Efficacy of TCZ in Protection of Radiographic Progression of Joints

In addition to clinical efficacy of TCZ in disease activity, TCZ showed beneficial effects in radiographic progression of joints (Table 1(c)). In the SUMURAI study, the TCZ group showed statistically significantly less radiographic change in the van der Heijde-modified Total Sharp Score (TSS) than the DMARD group at week 52 [69]. Moreover, the LITHE trial proved that at 52 week, the TCZ (either 4 mg/kg or 8 mg/kg) plus MTX group showed less progression of joint damage than the MTX group, as evaluated with the Genant-modified TSS (GmTSS) method [67].

### 5.4. Efficacy of TCZ in Phase IIIb/IV Trials and Clinical Practice

Following the seven phase III clinical trials, several phase IIIb/IV studies were conducted. The REACTION study performed in Japan showed that by 24-week treatment with TCZ, average disease activity score (DAS) 28 of 229 patients significantly decreased from 5.70 to 3.25 and a European League Against Rheumatism (EULAR) good response and DAS remission was achieved in 57.4% and 40.7% of the patients, respectively [71]. Moreover, at week 52, radiographic nonprogression and functional remission were achieved in 62.8% and 26.4% of 232 patients, respectively [72]. Interestingly, progression of joint destruction was found to be similar with or without concomitant MTX, glucocorticoids, or previous use of TNF inhibitors. The ACT-RAY trial was performed to compare TCZ plus MTX with TCZ plus a placebo in a setting that closely resembled real-life clinical practice [73]. After 24 weeks, ACR20, 50, and ACR70 response rates were 71.5%, 45.5%, and 24.5%, respectively, for the TCZ plus MTX group and corresponding rate of 70.3%, 40.2%, and 25.4% for the TCZ monotherapy group. This study demonstrated that TCZ plus MTX combination therapy and TCZ monotherapy could both be expected to be effective in real-life clinical practice, and importantly, that TCZ plus MTX combination was not significantly superior to TCZ monotherapy (Table 2). These and other studies showed that TCZ treatment improved disease activity, joint destruction, and quality of life. Moreover, a recent trial comparing TCZ (8 mg/kg intravenously every 4 weeks) monotherapy with adalimumab (40 mg subcutaneously every 2 weeks) monotherapy (ADACTA trial) proved the clinical superiority of TCZ [74] (Table 2). TCZ as monotherapy can thus be considered to be more beneficial than other biologics [75]. However, a meta-analysis of systematic reviews of clinical trial data indicates that TCZ, TNF inhibitors, and abatacept have similar efficacy in combination with MTX [76].

### 5.5. Efficacy of Subcutaneous Injection of TCZ in Phase III Trials

Intravenous injection every 4 weeks of TCZ (4 or 8 mg/kg) is currently used for the treatment of moderate-to-severe active RA, but recent clinical trials (MUSASHI and SUMMACTA) demonstrated that subcutaneous administration of TCZ (162 mg) weekly or every 2 weeks showed efficacy and safety comparable to those of intravenous injection of TCZ (8 mg/kg every 4 weeks) [77, 78] (Table 2). The MUSASHI study was a double-blind, double-dummy, parallel-group, comparative phase III study to evaluate the efficacy and safety of subcutaneous (SC) versus intravenous (IV) TCZ monotherapy for patients with RA and an inadequate response to synthetic DMARDs and/or biologics. A total of 346 patients were randomized to receive TCZ-SC 162 mg every 2 weeks or TCZ-IV 8 mg/kg every 4 weeks. At week 24, ACR20 response was achieved in 79.2% of the TCZ-SC group and in 88.5% of the TCZ-IV group, showing that TCZ-SC was not inferior to TCZ-IV [77]. The incidences of all adverse events (AEs) and serious AEs were 89.0% and 7.5% for the TCZ-SC group and 90.8% and 16.4% for the TCZ-IV group, respectively, while serum trough TCZ concentrations were similar for the two groups during the test period. The SUMMACTA trial was a randomized, double-blind, parallel-group study to evaluate the safety and efficacy of TCZ-SC in comparison with TCZ-IV combined with DMARD for patients with moderate-to-severe RA. A total of 1,262 patients were randomly assigned to receive TCZ-SC 162 mg weekly or TCZ-IV 8 mg/kg every 4 weeks in combination with DMARD [78]. At week 24, 69.4% of the TCZ-SC-treated patients versus 73.4% of the TCZ-IV-treated patients attained an ACR20 response. Moreover, ACR50/70 responses, DAS28 improvement and the safety profiles were similar for the two groups.

## 6. Safety Profile of Tocilizumab

The comparison of AEs between the control population (4,199) and the TCZ-treated population (4,009) was reported in 2011 [79]. Overall AE and serious AE rates were 278.2/100 patient-year (PY) and 14.4/100 PY, respectively. These events included serious infections (4.7/100 PY), opportunistic infections (0.23/100 PY), gastrointestinal perforations (0.28/100 PY), malignancy (1.1/100 PY), myocardial infarction (0.25/100 PY), and stroke (0.19/100 PY). Short-term (28 weeks) safety of TCZ for 7,901 patients was monitored in a postmarketing surveillance in Japan [80]. The incidence of total AEs and serious AEs was 43.9% and 9.6%, respectively. Infection and infestation were the most frequent AEs (11.1%) and serious AEs (0.5%). Analysis of long-term safety showed that rates of serious AEs, serious infections, and cardiovascular events remained stable during continued exposure to TCZ in long-term clinical trials. Infection was identified as the most frequent serious AE. The most commonly reported infections in RCTs were pneumonia (0.9/100 PY) and skin or soft tissue infections (0.9/100 PY). These results lead to the conclusion that infections were the most frequent AEs but a meta-analysis comparing the safety profile of TCZ with that of other biologics including TNF inhibitors, anakinra (IL-1R antagonist), abatacept, and rituximab showed similar rates of infection [81]. In contrast to the finding for infections, no increase in the incidence of malignancy or reactivation of tuberculosis was seen in TCZ-treated RA patients [82]. Gastrointestinal perforation appeared to be an AE specific for TCZ with an incidence rate of 1.9/1,000 PY [83]. This rate fell between those of 3.9/1,000 PY for corticosteroids and 1.3/1,000 PY for TNF inhibitors listed in the United Health Care database. While it is not clear at present why IL-6 blockade induced perforation, most cases were complications of diverticulitis. IL-6 also affects metabolism. Increases in mean fasting levels of plasma lipids such as total cholesterol, low-density lipoprotein, triglycerides, and high-density lipoprotein were detected in 20–30% of patients treated with TCZ. These higher lipid levels resulting from TCZ treatment are perhaps mediated by the influence of TCZ on lipoprotein receptor expression, since it has been recently shown that overproduction of IL-6 lowers blood lipid levels via upregulation of the very-low-density lipoprotein (VLDL) receptor [84]. In spite of this elevation of lipids, an analysis combining the data of various clinical trials showed no apparent increase in cardiac events in a followup of up to 5 years [82].

## 7. Other IL-6 Inhibitors in Development

The success of the indication of TCZ for the treatment of RA clarified that IL-6 blockade was a therapeutic strategy for RA, so that other IL-6 inhibitors are now being developed. These include fully human anti-IL-6R Ab (sarilumab/REGN88/SAR153191), anti-IL-6R nanobody (ALX-0061), anti-IL-6 Abs such as sirukumab (CNTO 136), BMS-945429 (ALD518), olokizumab (CDP6038), and MEDI5117, and soluble gp130-Fc fusion protein (FE301), which selectively inhibits trans-signaling but not classic signaling [5].

The favorable results of phase II, randomized, double-blind, placebo-controlled trials of sarilumab [85] and sirukumab [86] confirmed the effectiveness of IL-6 blockade strategy in RA. The phase II MOBILITY study evaluated efficacy and safety of subcutaneous injection of sarilumab, in which 306 RA patients were randomized to receive a 12-week administration of sarilumab 100 mg or 150 mg every week, 100 mg, 150 mg, or 200 mg every 2 weeks, or placebo added to stable MTX [85]. An ACR20 response was seen in 49.0% of the patients receiving the lowest sarilumab dose regime and in 72.0% of the patients receiving the highest dose regime, compared to 42.0% of those treated with placebo plus MTX. The types and incidence of AEs were consistent with those previously reported for TCZ. Sirukumab is a fully human monoclonal Ab to IL-6, and 151 RA patients were enrolled into a phase II trial [86]. The patients were randomized equally to receive subcutaneous injections of placebo every 2 weeks for weeks 0–10 and sirukumab 100 mg every 2 weeks for weeks 12–24, or sirukumab 25, 50, or 100 mg every 4 weeks, or 100 mg every 2 weeks for weeks 0–24. At week 12, more patients receiving sirukumab were in remission than those given the placebo according to Boolean- and simplified disease activity index (SDAI)-based ACR/EULAR criteria (2% versus 0% and 6% versus 3%). At week 24, high remission rates were attained with sirukumab at dose regimens ranging from 25 to 100 mg every 2–4 weeks, determined with ACR/EULAR or DAS28 (CRP) criteria. The types and incidence of AEs were consistent with those observed for TCZ.

## 8. Perspectives

In view of the outstanding clinical efficacy and tolerable safety of TCZ, TCZ is now recommended as one of first-line biologics for the treatment of active RA. However, several issues need to be clarified for realization of the optimal use of TCZ. First, an important issue is to clarify the mechanisms, which render IL-6 blockade efficacious for RA. Although it is clear that TCZ treatment led to improvements in markers related to systemic inflammation and bone and cartilage metabolisms [87–89], it remains to be determined whether the treatment can correct fundamental immunological abnormalities in RA [90]. As mentioned before, IL-6 has the capability of promoting autoantibody production and of causing imbalance between Th17 and Treg [31, 32]. Recent preliminary studies showed that TCZ treatment could rectify the imbalance in the peripheral blood CD4+ T cell population [91, 92]. Moreover, a 6-month treatment with TCZ led to a selective decrease in IL-21 production by memory/activated T cells in eight patients with RA [93]. Elevation of IL-21 has been detected in patients with RA [94] and is known to induce plasma cell differentiation and induce IgG4 production but the TCZ treatment resulted in a reduction in IgG4 subclass ACPA titer [35, 94]. These findings suggest that IL-6 blockade strategy may indeed correct immunological abnormalities in RA, but the findings of these studies have limited robustness due to the small sample size, so that further analyses will be required.

Second, the reason or reasons why IL-6 synthesis is continuously induced in RA remain to be clarified. One genetic polymorphism (−174) in the IL-6 gene promoter, which was found to affect IL-6 levels [95], did not appear to universally increase susceptibility to RA, but a recent meta-analysis showed that the −174 polymorphism might confer susceptibility to RA, at least in Europeans [96]. IL-6 can be produced by immune competent cells, fibroblasts, synoviocytes, endothelial cells, and many other cells in response to various stimuli [13]. The synthesis of IL-6 is strictly regulated by transcriptional and posttranscriptional mechanisms and a number of transcriptional factors, RNA binding proteins, and microRNAs have been shown to control IL-6 synthesis [97]. Moreover, it has been recently reported that newly found molecules such as Regnase-1 and Arid5a affect posttranscriptional regulation of IL-6 mRNA degradation [98–100]. Regnase-1 binds to the 3′ untranslated region of IL-6 mRNA and splits up IL-6 mRNA, whereas Arid5a binds to a similar region and stabilizes IL-6 mRNA. Moreover, some viral proteins or microRNAs reportedly activate the IL-6 gene and/or inhibit mRNA degradation [97]. It can therefore be anticipated that clarification of mechanisms by which dysregulated, persistent production of IL-6 is induced in RA will lead to an enhanced understanding of the pathogenesis of RA.

## Figures and Tables

**Table:** (a) Clinical efficacy of tocilizumab (Tocilizumab combination therapy)

Study	Population	Week at evaluation	Treatment arms	Patient number	HAQ (% ≥MCID)	Response rates (%), OR (95% CI)			DAS28 < 2.6 remission rate (%), OR (95% CI)
						ACR20	ACR50	ACR70	
TOWARD	DMARDs-IR	24 W	TCZ (8 mg/kg) + DMARDs	803	60****	61****	38****	21****	30****, 13.8
			DMARDs	413	34	25	9	3	3

RADIATE	Anti-TNF-IR	24 W	TCZ (4 mg/kg) + MTX	161	Δ − 0.3**	30***	17****	5	8, 4.3
			TCZ (8 mg/kg) + MTX	170	Δ − 0.4****	50***	29****	12****	30***, 21
			MTX	158	Δ − 0.1	10	4	1	2

OPTION	MTX-IR	24 W	TCZ (4 mg/kg) + MTX	214	Δ − 0.52*	48****, 2.6 (1.7–3.9)	31****, 3.8 (2.3–6.5)	12****, 7.0 (2.4–20.4)	13***, 18.8 (2.5–142)
			TCZ (8 mg/kg) + MTX	205	Δ − 0.55**	59****, 4.0 (2.6–6.1)	44****, 6.6 (3.9–11.2)	22****, 14.2 (5.0–40.4)	27****, 45 (6.1–332)
			MTX	204	Δ − 0.34	26	11	2	1

LITHE	MTX-IR	52 W	TCZ (4 mg/kg) + MTX	399	60	47*	29*	16*	30*, 4.92
			TCZ (8 mg/kg) + MTX	398	63*	56****	36****	20****	47****, 10.2
			MTX	393	53	25	10	4	8

**Table:** (b) Tocilizumab monotherapy

Study	Population	Week at evaluation	Treatment arms	Patient number	HAQ (% ≥MCID)	Response rates (%), OR (95% CI)			DAS28 < 2.6 remission rate (%), OR (95% CI)
						ACR20	ACR50	ACR70	
AMBITION	MTX, anti-TNF naïve	24 W	TCZ (8 mg/kg)	286	Δ − 0.7	70***	44**	28***	34^n.d.^, 5.83 (3.27–10.4)
			MTX	284	Δ − 0.5	53	34	15	12

SAMURAI	DMARDs-IR	52 W	TCZ (8 mg/kg)	157	68***	78***	64***	44***	59***, 46.5
			DMARDs	145	40	34	13	6	3

SATORI	MTX-IR	24 W	TCZ (8 mg/kg)	61	67****	80***	49^n.d.^	30^n.d.^	43***, 37.0
			MTX	64	34	25	11	6	2

*P < 0.05, **P < 0.01, ***P < 0.001, ****P < 0.0001.

HAQ: health assessment questionnaire disability index; MCID: minimal clinical important difference; OR: odds ratio; CI: confidence interval; DMARDs: disease-modifying antirheumatic drugs; IR: inadequate response; TCZ: tocilizumab; TNF: tumor necrosis factor; MTX: methotrexate; n.d.: not described.

**Table:** (c) Efficacy of tocilizumab in protection of radiographic progression of joints

Study	Radiographic assessment	Week at evaluation	Treatment arms	Proportion without progression TSS ≦ 0	Change in score (95% CI)		
					Total score	Erosion score	JSN score
SAMURAI	van der Heijde-modified Sharp score	52 W	TCZ (8 mg/kg)	56**	2.3**, (1.5–3.2)	0.9***, (0.3–1.4)	1.5*, (0.9–2.1)
			DMARDs	39	6.1 (4.2–8.0)	3.2 (2.1–4.3)	2.9 (2.0–3.8)

LITHE	Genant-modified Sharp score	52 W	TCZ (4 mg/kg) + MTX	81****	0.34****	0.21*	0.13*
			TCZ (8 mg/kg) + MTX	84****	0.29****	0.17****	0.12**
			MTX	67	1.13	0.71	0.42

*P < 0.05, **P < 0.01, ***P < 0.001, ****P < 0.0001.

TSS: total Sharp score; CI: confidence interval; TCZ: tocilizumab; DMARDs: disease-modifying antirheumatic drugs; JSN: joint space narrowing; MTX: methotrexate.

**Table 2:** Pivotal clinical trials of tocilizumab.

Study	Population	Week at evaluation	Treatment arms	Patient number	HAQ (% ≥MCID)	Response rates (%), OR (95% CI)			DAS28 remission rate (%), OR (95% CI)	Conclusion
						ACR20	ACR50	ACR70		
ACT-RAY	MTX-IR	24 W	TCZ (8 mg/kg) + PBO	276	Δ − 0.5	70	40	25	35	No difference of efficacy between TCZ and TCZ + MTX
			TCZ (8 mg/kg) + MTX	277	Δ − 0.5	72	46	25	40, 5.6 (−2.4–13.7)	

ADACTA	MTX-IR	24 W	TCZ-IV (8 mg/kg/4 weeks)	163	Δ − 0.7	65** 2.0 (1.2–3.1)	47*** 2.4 (1.5–3.9)	33** 2.3 (1.3–3.8)	40**** 5.7 (3.1–10.3)	TCZ is superior to ADA as monotherapy
			ADA-SC (40 mg/2 weeks)	162	Δ − 0.5	49	28	18	11	

MUSASHI	MTX-IR	24 W	TCZ-IV (8 mg/kg/4 weeks)	173	68	89	67	41	62	Noninferiority of TCZ-SC to TCZ-IV
			TCZ-SC (162 mg/2 weeks)	173	57	79	63	37	50	

SUMMACTA	DMARDs-IR	24 W	TCZ-IV (8 mg/kg/4 weeks) + DMARD	631	67	73	48	27	36	Noninferiority of TCZ-SC to TCZ-IV
			TCZ-SC (162 mg/week) + DMARD	631	65	69	47	24	38	

**P < 0.01, ***P < 0.001, ****P < 0.0001.

HAQ: health assessment questionnaire disability index; MCID: minimal clinical important difference; OR: odds ratio; CI: confidence interval; MTX: methotrexate; IR: inadequate response; TCZ: tocilizumab; PBO: placebo; IV: intravenous injection; ADA: adalimumab; SC: subcutaneous injection; DMARDs: disease-modifying antirheumatic drugs.
